# Adherence to antiretroviral treatment in 2013 between myth and reality

**DOI:** 10.1186/1471-2334-13-S1-P5

**Published:** 2013-12-16

**Authors:** Alina Cibea, Mariana Mărdărescu, Ruxandra Neagu-Drăghicenoiu, Cristina Petre, Rodica Ungurianu, Sorin Petrea, Ana Maria Tudor, Delia Vlad, Carina Matei

**Affiliations:** 1 National Institute for Infectious Diseases “Prof. Dr. Matei Balş” Bucharest, Romania

## Background

The number of children born to HIV seropositive mothers in Romania has increased in the past few years, since the patients from the ’90s cohort have reached reproductive age and new cases of sexually or drug infected women have emerged.

## Case report

We present the case of a 9 year, 6 month old girl, diagnosed with HIV at the age of 2, right after the death of her HIV seropositive father (later the mother was also diagnosed with HIV). For more than 7 years the mother did not administer the child the antiretroviral treatment that she was supplied with. In January 2013, after the mother’s death, the patient’s health worsened. She was admitted for multiple opportunistic associated infections, but, despite the complex therapeutic treatment administered, the evolution was not favorable.

## Conclusion

Adherence to medical care and antiretroviral therapy of a seropositive child is still a challenge and it actually involves the adherence of both parent and child. Because of prolonged treatment abandonment, the management of HIV infection associated with severe opportunistic infections is extremely difficult, due to lack of treatment response of an exhausted organism, limited therapeutic options, drug interactions, and adverse events.

